# Impacts of larval host plant species on dispersal traits and free-flight energetics of adult butterflies

**DOI:** 10.1038/s42003-022-03396-8

**Published:** 2022-05-16

**Authors:** Victoria M. Pocius, Staci Cibotti, Swayamjit Ray, Obenewa Ankoma-Darko, Nathaniel B. McCartney, Rudolf J. Schilder, Jared G. Ali

**Affiliations:** 1 Department of Biological Sciences, The University of Alabama, Tuscaloosa, AL USA; 2 Department of Entomology, The Pennsylvania State University, University Park, PA USA; 3 Department of Plant Pathology, Cornell University, Ithaca, NY USA

**Keywords:** Ecophysiology, Evolutionary ecology

## Abstract

Animals derive resources from their diet and allocate them to organismal functions such as growth, maintenance, reproduction, and dispersal. How variation in diet quality can affect resource allocation to life-history traits, in particular those important to locomotion and dispersal, is poorly understood. We hypothesize that, particularly for specialist herbivore insects that are in co-evolutionary arms races with host plants, changes in host plant will impact performance. From their coevolutionary arms-race with plants, to a complex migratory life history, Monarch butterflies are among the most iconic insect species worldwide. Population declines initiated international conservation efforts involving the replanting of a variety of milkweed species. However, this practice was implemented with little regard for how diverse defensive chemistry of milkweeds experienced by monarch larvae may affect adult fitness traits. We report that adult flight muscle investment, flight energetics, and maintenance costs depend on the host plant species of larvae, and correlate with concentration of milkweed-derived cardenolides sequestered by adults. Our findings indicate host plant species can impact monarchs by affecting fuel requirements for flight.

## Abstract (web summary)

The growth of muscle and flight performance in monarch butterflies is influenced by the plant species the larvae grow on.

## Introduction

Significant progress in our mechanistic understanding of plant-insect herbivore interactions has been made since Ehrlich & Raven^1^ first recognized their coevolutionary nature. Much of this work has focused on host plant interactions with larval insects^2,3^. This is not surprising since numerous plant-herbivore systems under scrutiny involve insect larvae that inflict significant damage to economically important (crop) plants. Effects of early-life nutrition on late-life metrics have been studied using approaches such as nutritional geometrical frameworks^4^. There has also been work on how larval host plant experiences may persist throughout ontogeny and affect adult traits^5–7^. However, little is known about how variation in larval host plant quality affects traits important to adult flight performance, which is key to the reproduction, dispersal and distribution dynamics of many insects. Larval food quality and availability have long been hypothesized to drive changes in dispersal capacity and/or propensity in adult insects^8^, and these factors have been demonstrated to affect morphological traits important to insect flight (reviewed in^9,10^). Yet, few studies have described effects of larval diet quality on actual flight performance in adults. Larval diet quality and quantity affected free-flight energy expenditure (*i.e*., flight metabolic rate) of adult *Manduca sexta* and *Spodoptera frugiperda*, respectively^7,11^, while, also in *M. sexta*, larval diet quality was shown to affect the lipid content of adult moths, and flying interactions with feeding arrays, although feeding frequency was unaffected^12^. Finally, in the migratory locust *Oedaleus asiaticus*, nymphs reared on high quality diet produced adults with enhanced migratory morphology (*i.e*., increased wing area, and mass allocation to the thorax and legs) and tethered flight activity^13^.

To further our understanding of this aspect of plant-insect herbivore interactions, we sought to examine how larval dietary history affects flight performance traits of adults in the monarch butterfly (*Danaus plexippus*), whose larval host plant interactions are a model system for chemical ecology- and coevolution research fields^1,14–16^.

Monarch butterfly populations rely primarily on the availability of milkweed (*Asclepias spp*.) to complete their annual life cycle, which includes a generation that migrates to-and-from winter roosting sites in Mexico and locations in the rest of North America^17,18^. While the common milkweed, *A. syriaca*, is distributed across their North American range, monarchs can encounter a variety of milkweed species as multiple generations of breeding females oviposit eggs during the northward migration^17^. Moreover, loss of suitable monarch habitat due to the expansion of residential and agricultural areas in the continental US, and increased herbicide and insecticide use^19–21^, have led to large-scale efforts to replant monarch migration corridors with a variety of milkweed species^22^.

Despite their specialist reputation, monarchs are sensitive to the variable defenses that different species of milkweed harbor^23–28^. Indeed, monarch larval growth rate varies more than ten-fold across different milkweed host species and is correlated with milkweed tissue content of sticky latex and cardenolides (i.e., toxic steroids)^29^, defensive compounds that are also inducible by herbivory^23^. Milkweed host species also was shown to affect monarch pupal, adult size, and lipid content^30^. Cardenolides disrupt Na^+^/K^+^-ATPase activity in cells^23,24^ and thus can exert considerable influence over chemical- and energy exchange processes in most species. While monarch Na^+^/K^+^-ATPase is considered relatively insensitive to cardenolide action^31^, this notion is primarily based on larval work focused on the cardenolide oubain, although several other cardenolides are present in milkweed for which Na^+^/K^+^-ATPase sensitivity is less well-understood. Furthermore, recent work has shown that variation levels of specific cardenolides can negatively impact monarch growth^32^. Overall, these findings indicate that milkweed host plants differ in their suitability to monarch larvae and may differentially affect the physiology adult monarchs.

Insect flight is an energetically costly trait. Flight muscle investment needs to be substantial to achieve body weight support aerodynamically and remain airborne (i.e., 12–65% of body mass^33,34^). Moreover, during free flight, insect flight muscle achieves respiration rates that are 20–100 fold (depending on the species) higher than those of resting insects^35^, these are the highest known respiratory rates (i.e., metabolic rates) for any locomotor tissue^36^. Therefore, factors that affect energy substrate storage, supply to-, and utilization by flight muscles, are expected to affect the flight performance of insects.

The study of monarch flight has been restricted largely to tracking migratory routes^37–39^, movement through local habitats^40^, and characterizing mechanisms of navigation^41–43^. Recently, however, Zhan et al.^44^ examined monarch flight from an evolutionary physiological and demographic perspective and demonstrated genetic differences between non-migratory (those never producing migrants) and migratory (those producing a long-distance migrant generation every year) monarch populations^44^. Importantly, this study showed that flight metabolic rate was significantly lower for non-migrant individuals from migratory populations than for those from non-migratory populations, suggesting that adult flight in the former is more energy efficient. Environmental factors contributing to variation in the cost of flight for adult monarchs have not yet been examined. While a recent study reported an effect of milkweed larval host species on adult wing morphology, adult flight distance and duration on flight mills was not affected by larval diet^45,46^. However, metabolic rates of insects “flying” in tethered preparations such as flight mills are significantly lower than those achieved during free flight^47–50^, presumably because there is no need to provide body weight support. Furthermore, given the diversity of plant defensive metabolites and the range of outcomes for insect development, studying how plant variation affects insect dispersal is vital to understanding this often-overlooked component of plant-herbivore ecology. Therefore, how larval dietary history may affect relevant costs of flight for adult monarchs remains unknown.

We hypothesized that monarch larval dietary history affects adult traits relevant to flight performance. Given that neural and excretory tissues in particular express high amounts of Na^+^/K^+^-ATPases and are significant contributors to energy expenditure at rest (i.e., resting metabolic rate)^51^, we also hypothesized that milkweed host plant species would affect resting metabolic rates of adult monarchs. To test these hypotheses, we reared *D. plexippus* larvae on eight *Asclepias* host species known to vary in toxicity level and ecological relevance (Table 1) and measured resting- and free-flight metabolic rates of resulting adults using flow-through respirometry. In addition, we examined host plant effects on adult flight muscle investment, energy stores, toxin sequestration and wing morphology (see Table S1 for full set of variables/results).

**Table 1:** Summary of milkweed species traits and ecological relevance.

Milkweed species	Common name	Native range	Foliar Cardenolide Content (% dry mass)	Evidence supporting ecological relevance
*A. curassavica*	Tropical Milkweed	Caribbean, Central and South America, Mexico	0.196	Non-native that disrupts migratory behavior^18^; common in gardens
*A. exaltata*	Poke Milkweed	Eastern Canada and United States	0.125	Low oviposition preference and high larval survival^25,94,95^; common in woodlands
*A. incarnata*	Swamp Milkweed	Northeastern and Southeastern United States	0.117	Preferred for oviposition with high larval survival, common in gardens^30,94–96^
*A. speciosa*	Showy Milkweed	Western North America	0.094	Slower larval growth rate compared to other species; common monarch larval host in Western United States^96,97^
*A. sullivantii*	Prairie Milkweed	Central North America	0.123	Similar oviposition preference to *A. syriaca*, but low larval survival^30,94,95^; cooccurs with *A. syriaca*
*A. syriaca*	Common Milkweed	Eastern North America	0.113	Most common larval host for migrant adults^71^
*A. tuberosa*	Butterfly Milkweed	United States	0.064	High larval survival, but low oviposition preference monarch host, common in gardens^30,94,95^
*A. verticillata*	Whorled Milkweed	United States	0.114	Low oviposition preference host^94,95^; common thin-leaved milkweed; broadly distributed.

Distribution information compiled from Woodson^91^ and NRCS and USDA^92^ Cardenolide information compiled from Agrawal et al.^93^.

## Results

**Fig. 1:**
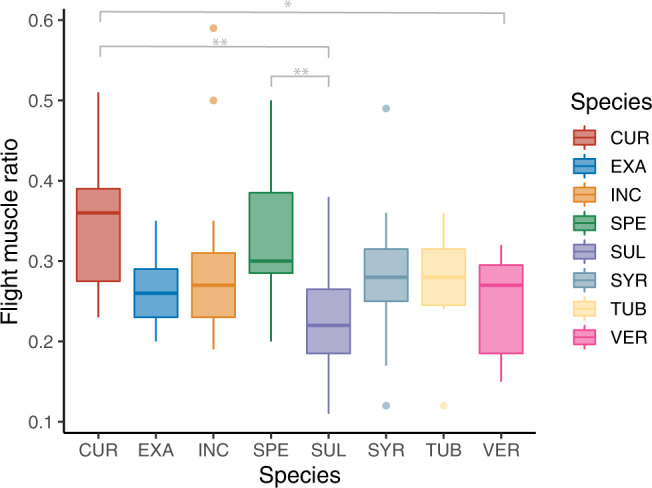
Adult flight muscle ratio as a function of larval host plant. Each box represents one milkweed species. CUR, A. curassavica; EXA, A. exaltata; HIR, A. hirtella; INC, A. incarnata; LAE, C. laeve; SPE, A. speciosa; SUL, A. sullivantii; SYR, A. syriaca; TUB, A. tuberosa; VER, A. verticillata; top horizontal bar in box represents third quartile, followed by median and first quartile; whiskers indicate data range. Connecting bars indicate significant post-hoc LS means comparisons (Tukey HSD).

Monarchs are a vagile species traveling up to 15 km per day during the breeding season^52^, and upwards of 250 m in only a few minutes^40^. To support this aerial performance it is expected that investment in flight muscle mass is significant^33^. Indeed, average flight muscle ratios – the percentage of total body mass consisting of flight muscle mass – of other *Danainae* butterflies are higher than 25%^53^. Our study showed that adult monarch flight muscle ratio was significantly affected by larval host plant (F_7,93_ = 4.61, *P* = 0.0002; Fig. 1). Specifically, mean flight muscle ratios of adults produced by larvae reared on tropical milkweed *A. curassavica* (35.4 ± 2.0%, *Ν* = 19) and showy milkweed *A. speciosa* (33.8 ±  2.2%, *Ν* = 15) were significantly higher (*cur-sul*: *P* = 0.0008, *cur-ver*: *P* = 0.017; *spe-sul*: *P* = 0.01, respectively; see Fig. 1) than those of adults reared on prairie milkweed *A. sullivantii* (22.5 ± 2.2%, *Ν* = 15) and whorled milkweed *A. verticillata* (24.2 ± 2.6%, *Ν* = 11).

**Fig. 2:**
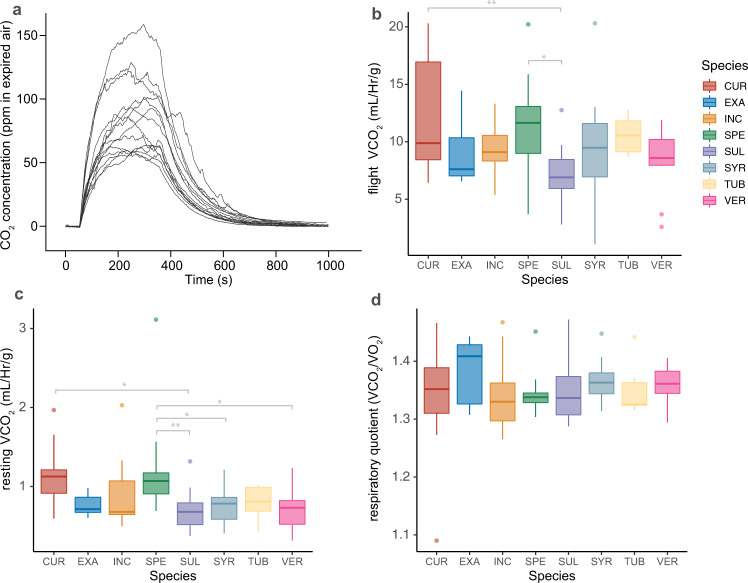
Results from respiration assays of Monarchs on each milkweed species. **a** Raw CO_2_ traces produced by monarchs flying in a respiratory chamber for 5 min; traces for adults reared on A. syriaca shown only. **b** Body mass normalized adult flight metabolic rate and **c** resting metabolic rate, and (**d**) respiratory quotient (RQ) as a function of larval host plant. CUR, A. curassavica; EXA, A. exaltata; HIR, A. hirtella; INC, A. incarnata; LAE, C. laeve; SPE, A. speciosa; SUL, A. sullivantii; SYR, A. syriaca; TUB, A. tuberosa; VER, A. verticillata; top horizontal bar in box represents third quartile, followed by median and first quartile; whiskers indicate data range. Connecting bars indicate significant post-hoc LS means comparisons (Tukey HSD).

Moreover, total body mass (F_7,121_ = 5.36, *P* < 0.0001) and forewing length (F_7,121_ = 4.19, *P* = 0.0004) varied significantly with larval host plant, while total body lipid content and other life history traits were unaffected (Table S2*)*. The observed increased flight muscle ratio for *A. curassavica-* and *A. speciosa*-derived adults may be a necessary consequence of their overall body mass increases and is generally predicted by unequal scaling of muscle performance and body size^51^. Importantly however, this extra flight muscle mass investment may also come at an increased energetic cost (per unit body mass) of flight. Indeed, mean normalized adult flight metabolic rate (FMR *i.e*., VCO_2_ during flight) was affected by larval diet (F_7,93_ = 3.09, *P* = 0.0056; Fig. 2b), as adults reared on *A. curassavica* and *A. speciosa* produced significantly higher FMR than those reared on *A. sullivantii* (*P* = 0.0031 and *P* = 0.044, respectively). Moreover, mean resting metabolic rate (RMR, *i.e*., VCO_2_ at rest) also varied significantly among larval dietary groups (F_7,93_ = 4.09, *P* = 0.0006; Fig. 2c). Specifically, adults reared on *A. speciosa* had a significantly higher normalized RMR than those reared on *A. sullivantii* (*P* = 0.0075)*, A. verticillata* (*P* = 0.0283), and *A. syriaca* (*P* = 0.0276), while those reared on *A. curassavica* had significantly higher normalized resting metabolic rate than *A. sullivantii* (*P* = 0.0181). These results indicate that in addition to energy expenditure during flight, larval milkweed host species affect the energetic maintenance costs of adult monarchs.

Using VCO_2_ as a proxy for energy expenditure during flight assumes that the respiratory quotient (i.e., VCO_2_/VO_2_, or flight muscle substrate use) stayed constant. While Lepidoptera have generally been observed to use carbohydrates as flight muscle fuels, respiratory quotients of adults in free flight have been reported to vary with dietary intake^51^. Here we report that larval milkweed diet did not affect the mean respiratory quotient of flying adult (Fig. 2d), indicating that the observed effects on flight metabolic rate in our study are indicative of changes in overall energy expenditure rather than diet-induced shifts in flight muscle substrate use. Thus, adults derived from larvae reared on *A. curassavica* required significantly more energy per unit body mass for flight than those reared on *A. sullivantii* and *A. verticillata*. Interestingly, mean monarch RQ values varied between 1.3–1.4 (Fig. 2d), which is similar to mean RQ values reported for *Manduca sexta* hawkmoths by Levin et al.^54^, who suggest these relatively high values (i.e., aerobic, pure carbohydrate oxidation should result in RQ values approximating 1.0) are likely caused by the use of the pentose phosphate shunt during flight activity in these nectivorous insects.

**Fig. 3:**
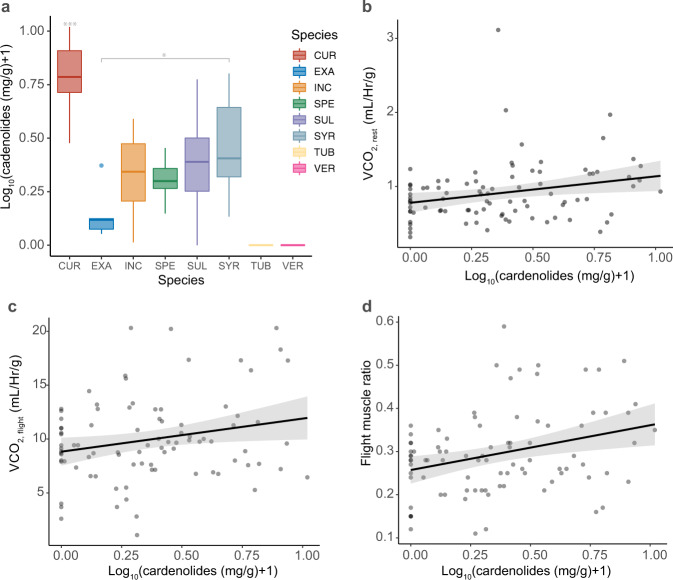
Interactions with cardenolides on sequestration, respiration and musculature. **a** Wing cardenolide content (mg/g) of adult monarchs by host plant; wing samples of individuals reared on TUB and VER did not contain cardenolides. Each box represents one milkweed species. CUR, A. curassavica; EXA, A. exaltata; HIR, A. hirtella; INC, A. incarnata; LAE, C. laeve; SPE, A. speciosa; SUL, A. sullivantii; SYR, A. syriaca; TUB, A. tuberosa; VER, A. verticillata; top horizontal bar in box represents third quartile, followed by median and first quartile; whiskers indicate data range. **b** Correlation of VCO_2_ Rest (mL/Hr/g) and adult wing cardenolide content (r^2^ = 0.064, F(1,86) = 5.84, *P* = 0.0178). **c** Correlation of VCO_2_ (mL/Hr/g) and adult wing cardenolide content (r^2^ = 0.050, F(1,86) = 4.56, *P* = 0.0357). Cardenolide values were log transformed prior to analysis. **d** Correlation of Flight muscle ratio and adult wing cardenolide (r^2^ = 0.099, F(1,86) = 9.41, *P* = 0.0029).

**Fig. 4:**
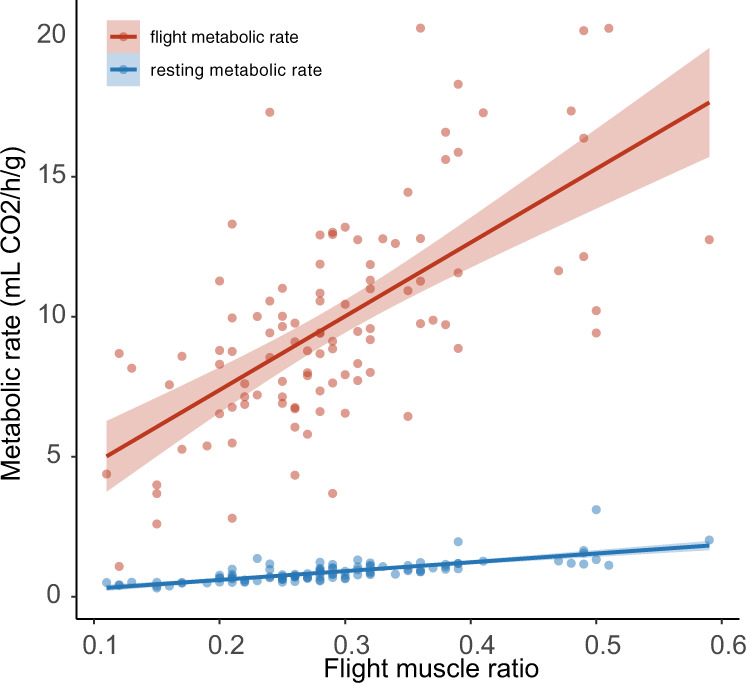
Correlations of flight muscle ratio and energy expenditure. (Blue) Correlation of VCO_2_ Rest (mL/Hr/g) and flight muscle ratio (r^2^ = 0.589, F(1,99) = 141.92, *P* = < 0.001) (Red) Correlation of VCO_2_ (mL/Hr/g) and flight muscle ratio (r^2^ = 0.418, F(1,99) = 70.97, *P* = < 0.001).

Although monarchs benefit from sequestration of milkweed cardenolides since they confer some level of protection against higher trophic levels^55,56^ and infection^57^, at high levels these toxins negatively affect larval growth and survival^23,29,31,55^. To start examining if sequestration-associated exposure to cardenolides may also affect adult energy expenditure, we extracted and quantified cardenolide content of wings from adult specimens used in the respirometry trials. We then examined how wing cardenolide content correlated with adult flight muscle mass ratio and observed flight and maintenance energetic costs. As expected from a previous report on milkweed cardenolide content^29^, the wings of adults reared on *A. curassavica* contained significantly higher amount of cardenolides than those reared on the other seven species (χ^2^ = 49.062, d.f. = 7, *P* = 2.206e^−8^, Fig. 3a). In addition, wing cardenolide content was a significant predictor of both normalized metabolic rate at rest and during flight (Fig. 3b, c), providing support for the hypothesis that larval exposure to higher levels of toxic host plant defenses (in the form of cardenolides) causes increased energy expenditure by adult butterflies. Although wing cardenolide content may not be the ultimate proxy for cardenolide exposure, it is correlated to the amount in the abdomen and thorax^58^ and an indicator for the amount they were exposed to in their host^29^. We recognize it is possible that milkweed host quality traits other than cardenolide content^28,59,60^ might have mediated the effects on adult traits observed in this study. For example, adults reared on *A. speciosa*, that demonstrated high flight metabolic rates (Fig. 2b), did not sequester significantly higher cardenolide levels than those reared on *A. syriaca* (Fig. 3a; *P* = 0.130; *A. syriaca* produced adults with intermediate flight metabolic rates) and *A. sullivantii* (Fig. 3a; *P* = 0.955; *A. sullivantii* produced adults with low flight metabolic rate), and significantly lower amounts than those reared on *A. currassavica* (Fig. 3a; *P* < 0.0001). In addition to the correlations of wing cardenolide content and flight muscle ratio (Fig. 3d), and maintenance- and flight energetic costs, we find that flight muscle ratio by itself explained 42 and 59% of variation in mass-specific flight- and resting metabolic rates, respectively (Fig. 4).

Prior work demonstrated that larval dietary history affects adult lipid storage in monarchs and other insects^6,30^, but our study showed no effects of milkweed host on adult lipid content (Table S1). This is likely because adult monarchs in these experiments had *ad libitum* access to carbohydrate-rich artificial nectar during the eight days prior to flight trials (see *Methods*). Monarchs typically eclose with a small, variable lipid store^30^, but larger lipid stores are needed to prompt reproductive development^61^ and support long distance dispersal flights^62^. Adult monarchs maintain and build upon their lipid stores throughout the breeding season^39,63,64^ by converting dietary carbohydrates to lipid as has been documented for other insects^65–69^. Thus, pre-flight trial carbohydrate intake likely allowed adults to supplement their lipid stores and mask any potential effects of larval diet on lipid content, in a fashion similar to that demonstrated for other butterflies that sought out nectar rich in amino acids to compensate for a poor larval diet^70^.

## Discussion

Overall, our study suggests that trophic interactions between larval insect herbivores and their host plants can affect key adult insect fitness traits such as flight muscle investment, maintenance- and flight energetics. Interestingly, tropical milkweed (*A. curassavica*), a rather controversial monarch host plant for monarchs in the migratory ranges^18^, produced larger adults with higher flight muscle investment (Fig. 1) that incur the highest energetic costs of flight for a given body mass (Fig. 2b). In comparison, common-, prairie- and whorled milkweed (*A. syriaca, sullivantii* and *verticillata*, respectively) produced relatively small adults with lower energetic costs of flight and maintenance (Fig. 2c). Although tropical milkweed (non-native to the U.S. and Canada) is not used in most replanting efforts to re-establish monarch habitat, its range has been increasing northward from Mexico and frequently overlaps with that of native milkweed host plant populations^18^. Common milkweed is by far the most common naturally occurring milkweed, supports development of wing shapes suited for long-distance flight^45^ and remains the most important plant species for the monarch populations that will undertake the longest southward migratory flights. Most monarch butterflies at Mexican overwintering sites feed on common milkweed as larvae^71^ and tropical milkweed is thought to interfere with monarch migration when it is planted as a resource to monarchs in migratory corridors by transitioning migrants to the reproductive stage early or the proliferation of pathogens^18,72–74^. Our study suggests that one contributor to this interference may be tropical milkweed’s effect on adult monarch energetics. Disentangling the relative contribution of nutrition, defenses, flight muscle would require additional studies focusing on just this species. Work by Agrawal et al.^32^ recently examined such mechanisms for larval performance on tropical milkweed.

Although relationships between milkweeds, monarchs, and toxins have played a central role in our understanding of coevolution, plant defense, sequestration, and animal behavior^17^, understanding the mechanistic link between host plant chemicals, sequestration and adult flight is challenging. There is some recent evidence that cardenolides can be a burden for monarchs and that there are costs to sequestration^32^. In vitro work by Petschenka et al.^75^ demonstrated that certain cardenolides can be strong inhibitors of neural function in monarchs. Thus, our work utilizing different milkweed species with unique cardenolide composition allows for potential costs to become apparent. There’s strong evidence suggesting monarchs selectively sequester more polar compounds, by either modification, detox, or transport^76^ and may even reduce exposure of larvae to more toxic compounds via ovipositional preference^32^. Thus, monarch mitigation strategies to sequester these poisonous compounds suggests costs not only to growth, survival, lifespan^77^, and here we find support for costs of sequestration that affects energetics as well.

The host plant effects on energetics may be primarily driven by their positive effects on animal size and flight muscle tissue investment. While increased size may generally convey higher quality in insects in terms of fecundity^78–80^, our findings underline that increased size also comes with relatively high energetic demands. Higher energetic costs for larger individuals may not be problematic in natural settings with ample energy resource availability to monarchs, but when energy availability is limited (*e.g*. during poor summer/fall conditions, availability of nectar resources) and/or when energy saving is of primary concern (*e.g*. during overwintering and long-distance dispersal) higher energetic demand may not be favored. Flight metabolic rates in monarchs from migratory populations were reported to be significantly lower than those from non-migratory populations in Southern Florida^44^, further supporting the idea that managing energy expenditure is a crucial trait to the dispersal and population dynamics of this species. Therefore, in particular if the energy expenditure of adult migrant phenotypes are even more sensitive to dietary history^13^ than non-migrant monarch phenotypes such as studied by Zhan et al.^44^ and in this study, milkweed larval host could negatively affect the ability of eastern US monarch migrants to reach Mexican roosts and overwinter successfully. Thus, laboratory work with migratory phenotypes and host plants should be done in future studies.

This study extends traditional approaches that have typically used a limited set of larval traits (*e.g*. larval growth and mortality) as overall measures of herbivore performance on host plants. Given the current lack of knowledge in this area and increasing availability of genomic resources in non-model systems, this work serves as an example for future studies in this and other systems aimed at achieving a mechanistic understanding of short- and long-term environmental variation on plant-herbivore interactions across life stages. With specific regard to the monarch butterfly, our findings contribute basic biological and ecological insights but may also inform efforts aimed at addressing monarch population declines. Here we find that efforts to protect a species, by selection of host plants, may have unintentional consequences that deserve further assessment. In particular, if larval milkweed host plants generally influence adult monarch energetic demands in significant ways, one of the key unanswered questions is how this may impact the yearly migratory journey many monarch populations undertake.

## Methods

### Plant and animal husbandry

Monarch eggs were obtained from a colony kept year-round at Iowa State University (ISU, USDA-ARS, Ames IA) for approximately 18 generations. Monarchs were collected in the early summer of 2018 (42 wild-caught adults) and added to the existing colony to prevent inbreeding. Only 3 generations of these mixed-collection adults preceded our study. Colony individuals were maintained on *A. syriaca* and *A. curassavica* seasonally at ISU. Eggs were laid on *A. curassavica* and shipped overnight on *A. curassavica* stems and eggs we received from this colony were the progeny of over 50 adult females; eggs from a specific female were not assigned to specific plant treatments. Eggs were transferred to an incubator kept at 25 °C, 16:8 L:D, 70% RH for 3 days until hatch. First instars were then transferred to milkweed plants to mature (See S1 for resulting sample sizes per plant species).

Eclosion date was recorded for each individual, and post eclosion, all adults were allowed to sclerotize in individual plastic cups for 24 h. Sclerotized adults were weighed to the nearest 0.01 mg in a Mettler AJ100 balance (Mettler-Toledo LLC, Columbus, OH, USA). Adult forewing length and hindwing length was measured to the nearest 0.01 mm with digital calipers (Neiko Tools, USA) and all adults were examined for the parasite *Ophryocystis elektroscirrha* (OE) using the tape method^81^. No adults used in respirometry assays (see below) were infected with OE. OE negative adult males and females were housed in separate mesh cages by milkweed species treatment to prevent mating. Adults were allowed to feed ad libitum from an artificial nectar solution containing 60 mg/mL sugars (glucose/dextrose mix), 4.18 mg/mL sodium (sodium citrate), and 0.13 mg/mL potassium (potassium phosphate) (Gatorade G series, lemon-lime flavor). Nectar dishes were changed every 72 h. Adults were allowed to feed for 8d before flight. Eight adults were excluded from free-flight respirometry assays due to wing damage or premature death.

Eight species of milkweed (*A. curassavica, A. exaltata, A. incarnata, A. speciosa, A. sullivantii, A. syriaca, A. tuberosa and A. verticillata*) were grown from seed at Pennsylvania State University without the use of chemical pesticides. Milkweed seed was obtained from Ernst Native Seed (*A. incarnata, A. speciosa, A. syriaca*, and *A. tuberosa;* Meadville, PA) or Prairie Moon Nursery (*A. exaltata, A. sullivantii*, and *A. verticillata*; Winona, MN). *A. curassavica* seeds were obtained through www.OutsidePride.com (Salem, Oregon, USA). All seeds were cold-stratified for 7d at 2.2 °C and moved to a 25 °C incubator (Caron 6030) set to 24:0L:D conditions until germination. Growing conditions were similar to those described in Pocius et al.^30^, but milkweeds were fertilized (Osmacote, Scotts Company LLC, Marysville, Ohio, USA) at 2w and 6w during our experiment. Seedlings were sown into 36-cell seedling trays (8.25 cm deep); 4–6w post-germination seedlings were transplanted into 8.9 cm square deep perennial pots (Kord, Ontario, Canada). Milkweeds were 9w old when offered to first instar monarch larvae. All plants were healthy with undamaged leaves at the start of the experiment.

### Larval feeding and survivorship

Five neonates were added to each plant inside a 40 × 40 × 60 cm mesh pop-up cage. We used a complete random block design with each block containing one cage of each milkweed species. There were 6 blocks of each milkweed species with the exception of *A. exaltata* (4 blocks) due to a limited number of healthy plants, for a total of 30 larvae per milkweed species (20 for *A. exaltata*) at the start of the experiment. All blocks were kept in the same greenhouse compartment, which was kept at ~25 °C, 16:8 L:D, 40% RH. Greenhouse temperature was recorded hourly via Thermocron sensors (Embedded Data Systems, iButton, New South Wales, Australia). Larvae were monitored for survivorship daily. Plants were watered daily, and new plants were added when the initial plants were defoliated. To reduce larval stress, we did not move larvae or take larval measurements. Larvae were only moved onto new plants when necessary until pupation. Cages were monitored for pupae starting at day 8 of the experiment. The date of pupation was recorded, and each pupa was allowed to sclerotize for 24 h before being carefully removed from each cage. Sclerotized pupae were weighed to the nearest 0.01 mg on a Mettler AJ100 balance (Mettler- Toledo LLC, Columbus, OH, USA). Pupal length and width were measured to the nearest 0.01 mm with digital calipers (Neiko Tools, USA). Individual pupae were attached to wooden skewers with small beads of hot glue and hung inside individual plastic cups for eclosion.

### Flow-through respirometry

Eight days post-eclosion, gas exchange of adults in free-flight was determined via flow-through respirometry. We used a 24 V DC circulation pump to push CO_2_-free air generated by a Whatman Ft-IR purge gas generator (Whatman International Ltd, Maidstone, UK) into two flow paths. Each path contained a clear 1 L plastic jar, but only one jar contained an adult monarch; the other path served as the reference (i.e., no monarch present) flow path. Flow rates were controlled using a Brooks 5850E mass flow controller (Brooks Instrument, Hatfield, PA, USA; calibrated by Coastal Instruments, Burgaw, NC, USA) and a FlowBar-8 system (Sable Systems, Las Vegas, NV, USA), for the path containing the animal jar and the reference jar, respectively. Incoming CO_2_-free air was scrubbed of residual CO_2_ and H_2_O using a Drierite/Ascarite/Drierite scrubber column prior to entry into jars. Both jars (and attached tubing) were placed in a Percival I-36VL incubator set to 28 °C; monarchs engage in spontaneous flight at 28 °C^82,83^ without pre-flight warm-up via shivering thermogenesis. Air leaving the jars flowed into two separate manifolds (50 mL syringe barrels) from which air was subsampled at 200 mL/min and pulled through a LI-COR 7000 (LI-COR Biosciences, Lincoln, NE, USA) CO_2_/H_2_O analyzer and a FC-2 Differential Oxygen Analyzer (Oxzilla II; Sable Systems, Las Vegas, NV, USA), using a SS4 subsampler (Sable Systems, Las Vegas, NV, USA). During flights, a mounted blacklight bulb was turned on inside the incubator as this facilitated continuous flight performance in preliminary trials.

Prior to introduction of a butterfly to the animal jar, the system was first completely flushed with CO_2_-free air. After introduction of a butterfly, the jar was covered with a black cloth to minimize activity and allow recording of resting metabolic rate. The cloth was then removed, and the jar was gently moved as needed to stimulate continuous flight. After 5 min, the jar was once again covered with the black cloth until the CO_2_ signal returned to a stable baseline. The full set of free-flight respirometry assays took multiple days to complete, so 8d-old butterflies reared on different milkweed species were selected randomly to avoid sampling bias and researchers were blind to monarch milkweed host. Adults were weighed immediately before each respirometry trial. After trials, wings were removed and stored at −80 °C. Abdomen and thorax mass were determined to the nearest 0.1 mg before being flash-frozen in liquid nitrogen and stored at −80 °C.

For calculations of resting- and flight metabolic rate, CO_2_ raw values (in ppm) first underwent a Z-transformation to recover approximate instantaneous gas exchange signals^84^, prior to being converted to emission rates (VCO_2_ in ml/Hr) using standard equations^85^. For calculations of respiratory quotients (RQ, i.e., VCO_2_/VO_2_), we used non-transformed CO_2_ and O_2_ traces because our O_2_ traces were significantly noisier than the CO_2_ signals. Resting metabolic rate was determined as the difference between the stable VCO_2_ of a butterfly at rest in the covered flight jar and the CO_2_-free air (prior to introduction of the butterfly). Flight metabolic rate was calculated by subtracting resting VCO_2_ from the average VCO_2_ across timepoints 50–250 s of the 5-minute flight bout (*i.e*., between ~0.8–4.2 min of flight). Data acquisition was performed using a UI2 interface and Expedata software (Sable Systems, Las Vegas, NV). CO_2_ and O_2_ raw trace conversions (and Z-transformations) and analyses were automated using a customized user interface developed with Igor Pro v8.02 software.

### Lipid assay

The dissected adult abdomen were dried at 60 °C for 24 h in a drying oven. The dried tissue was then weighed, placed into perforated gelatin capsules, and total lipids were extracted during a 48 h period using refluxing petroleum ether in a Soxhlet extractor apparatus^86–88^. After extraction, each abdomen was dried again for 24 h in a drying oven. Lean abdominal dry mass was subtracted from pre-extraction abdominal dry mass to obtain total tissue fat content.

### Cardenolide assay

To evaluate the relative abundance of cardenolides sequestered by caterpillars reared on the different milkweed species, adult wings were dried (at 50 °C) and ground. 21–76 mg of ground tissue, spiked with 20 μg of digitoxin as internal standard, was extracted with 1.8 mL of methanol in a sonicating water bath at 55 °C for 20 min. Wing cardenolides correlate tightly with body cardenolide concentrations^46,89^. After evaporating the solvent, the residue was resuspended in 0.5 mL methanol. Samples were analyzed by HPLC using a Zorbax StableBond C18 reversed phase column (5 μm, 150 × 4.6 mm, Agilent Technologies, Santa Clara, CA, USA) and an Agilent 1100 series instrument with diode array detection. The 15 uL injection was eluted at a constant flow of 0.7 mL/min with a gradient of acetonitrile and water as follows: 0–2 min 16% acetonitrile; 25 min 70% acetonitrile; 30 min 95% acetonitrile with a final 8 min hold. Peaks were detected by a diode array detector at 218 nm, and absorbance spectra were recorded from 200–400 nm. Peaks showing a characteristic symmetrical absorption band with a maximum between 217–222 nm were recorded as cardenolides^90^. Sample concentrations were quantified by relating abundances to the peak area of the internal standard.

### Statistical analyses

Data were analyzed using R version 3.5.3 (R Core Team 2014). Within each experiment, data were combined across trials (6 blocks total), as blocks were not significantly different from one another. Survivorship differences were determined using a log-rank test on the Kaplan–Meier survival estimates for larvae reared on each milkweed species. Pairwise log-rank tests were used to examine effects of species as this allowed us to include individuals that spent different amounts of time as larvae and pupae. A Bonferroni correction was used to adjust the significance level for pairwise comparisons (adjusted α = 0.0014). A one-way ANOVA was used to test for effects of larval diet on adult traits (i.e., body mass, forewing length, hindwing length, larval duration, pupal duration, thoracic mass, and abdominal lipid content), followed by Tukey HSD tests were used to assess pairwise differences. Flight muscle ratio was calculated as the ratio of thoracic mass and body mass. Flight muscle ratio values were arcsine transformed and effects of larval diet were assessed using one-way ANOVA and a Tukey HSD test to assess pairwise differences. Effects of larval diet on total wing cardenolide content were examined using a Kruskal-Wallis rank sum test, and a Dunn test for multiple comparisons was used to assess pairwise differences. Effects of larval diet on body mass-normalized average resting metabolic rate, -average free-flight metabolic rate, and average respiratory quotient were examined using one-way ANOVA, followed by Tukey HSD tests to assess pairwise differences. Spearman correlations were used to determine relationships between body mass-normalized average free-flight metabolic rate and average peak flight metabolic rate with total cardenolide content. Sexes were pooled for all analyses, as there were no significant differences when males and females were analyzed separately.

### Reporting summary

Further information on research design is available in the Nature Research Reporting Summary linked to this article.

## Supplementary information

Supplementary Information

Reporting Summary

## Data Availability

All data are available from the corresponding author(s) on reasonable request.
