# Supplementary material for: Impacts of larval host plant species on dispersal traits and free-flight energetics of adult butterflies
Source: Commun Biol. 2022 May 16;5:469. doi: 10.1038/s42003-022-03396-8 (PMC9110344; doi:10.1038/s42003-022-03396-8)
Supplement: Supplementary file 1 — Supplementary Information [file 42003_2022_3396_MOESM1_ESM.pdf]

Table S1.

Summary of Adult physical traits from butterflies reared on eight milkweed species and wild-caught migrant adults. Entries in the same column that do not share a letter are significantly different from each other. Data is displayed as the mean ± standard error. *N represents the number of monarchs that survived to the adult stage. For FWL, Percent Survival, and Lipid Content, we did not find any significant differences; these columns are not designated with letter markers within columns.

Milkweed Species	N*	Age (day)	Preflight Adult Mass (g)	FWL (mm)	HWL (mm)	Percent Surviva l to Adult	Dried Abdominal Mass (g)	Lipid Content (mg)	Lipid Content (% abdominal mass)	Thoracic Mass	Flight Muscle Ratio	Wing Cardenolide Content (mg/g
A. curassavica	22	8-10	0.572±0.02 ^{A}	47.74±0.67	32.67±0.71 ^{A}	73.3	0.05±0.003	12.3 ± 2.3	22.6	0.169±0.007	0.354±0.019^{A}	62.57±2.02^{A}
A. exaltata	8	8-10	0.526±0.08 ^{AB}	47.40±1.02	31.25±0.56 ^{AB}	40	0.04± 0.004	10.3± 3.4	23.4	0.142±0.009	0.262±0.022^{AB}	22.0±511^{BC}
A. incarnata	16	8-10	0.498± 0.02 ^{AB}	44.96±0.91	30.046±0.48 ^{B}	50	0.04±0.004	12.0 ± 2.7	24.1	0.150±0.009	0.298+0.035^{AB}	28.58±5.28^{B}
A. speciosa	17	8-10	0.506± 0.02 ^{AB}	44.66±0.85	30.18±0.82 ^{B}	60	0.05±0.004	17.1 ± 2.4	32.1	0.159±0.006	0.349±0.028^{A}	37.67±5.70^{B}
A. sullivantii	21	8-10	0.520± 0.04 ^{AB}	45.43±0.95	30.37±0.79 ^{B}	70	0.03±0.003	7.6 ± 1.4	19.7	0.124±0.009	0.230±0.019^{B}	45.85±13.5^{A}
A. syriaca	17	8-10	0.526± 0.02^{AB}	45.77±0.812	31.29±0.61 ^{AB}	63.33	0.04±0.003	10.1 ± 2.2	26.7	0.143±0.009	0.261±0.018^{A}	38.0±4.56^{B}
A. tuberosa	11	8-10	0.578± 0.06 ^{A}	46.35±0.95	30.57±0.61 ^{AB}	36.67	0.04±0.006	7.44 ± 2.3	19.7	0.144±0.015	0.268±0.029^{AB}	0^{C}
A. verticillata	17	8-10	0.475± 0.02 ^{B}	44.81±0.70	29.42±0.63 ^{B}	56.67	0.04±0.003	10.3 ± 2.1	26.5	0.123±0.011	0.238±0.023^{AB}	0^{C}
